# Anxiety and depression symptoms in adolescents and young adults with juvenile idiopathic arthritis: results of an outpatient screening

**DOI:** 10.1186/s13075-024-03312-x

**Published:** 2024-04-10

**Authors:** Florian Milatz, Jens Klotsche, Martina Niewerth, Claudia Sengler, Daniel Windschall, Tilmann Kallinich, Frank Dressler, Ralf Trauzeddel, Reinhard W. Holl, Ivan Foeldvari, Normi Brück, Svenja Temming, Toni Hospach, Petra Warschburger, Rainer Berendes, Gabriele Erbis, Jasmin B. Kuemmerle-Deschner, Frank Weller-Heinemann, Johannes-Peter Haas, Annabel S. Müller-Stierlin, Agnes Mutter, Thomas Meissner, Harald Baumeister, Kirsten Minden

**Affiliations:** 1 Programme area Epidemiology and Health Services Research, Deutsches Rheuma-Forschungszentrum Berlin, ein Institut der Leibniz-Gemeinschaft, Charitéplatz 1, 10117 Berlin, Germany; 2 Clinic of Paediatric and Adolescent Rheumatology, Northwest German Centre for Rheumatology, St. Josef- Stift Sendenhorst, Sendenhorst, Germany; 3 Department of Paediatric Respiratory Medicine, Immunology and Critical Care Medicine, Charité – Universitätsmedizin Berlin, corporate member of Freie Universität Berlin and Humboldt - Universität zu Berlin, Berlin, Germany; 4 Department of Paediatric Pneumology, Allergology and Neonatology, Children’s Hospital, Hannover Medical School, Hannover, Germany; 5 Department of Paediatrics, Paediatric and Adolescent Rheumatology, Helios Klinik Berlin-Buch, Berlin, Germany; 6 Institute for Epidemiology and Medical Biometry, ZIBMT, University of Ulm, Ulm, Germany; 7 Hamburg Centre for Paediatric and Adolescent Rheumatology, Schön Klinik Hamburg Eilbek, Hamburg, Germany; 8 Department of Paediatrics, Carl Gustav Carus, University Hospital, Technical University Dresden, Dresden, Germany; 9 Department of Paediatrics, Olgahospital, Klinikum Stuttgart, Stuttgart, Germany; 10 Department of Psychology, University of Potsdam, Potsdam, Germany; 11 Pediatric Rheumatology, Children’s Hospital St. Marien, Landshut, Germany; 12 Division of Pediatric Rheumatology and autoinflammation reference centre Tuebingen (arcT), Department of Pediatrics, University Hospital Tuebingen, member of ERN-RITA, Tuebingen, Germany; 13 Department of Pediatrics and Adolescent Medicine, Pediatric Rheumatology, Eltern-Kind-Zentrum Prof. Hess, Klinikum Bremen-Mitte, Bremen, Germany; 14 German Centre for Paediatric and Adolescent Rheumatology, Garmisch-Partenkirchen, Germany; 15 Department of Psychiatry and Psychotherapy II, Ulm University, Ulm, Germany; 16 Department of Clinical Psychology and Psychotherapy, Institute of Psychology and Education, Faculty of Engineering, Computer Science and Psychology, Ulm University, Ulm, Germany; 17 Department of General Paediatrics, Neonatology and Paediatric Cardiology, Medical Faculty, University Hospital Düsseldorf, Heinrich-Heine-University, Düsseldorf, Germany; 18 Medizinische Fakultät, Universität Halle-Wittenberg, Halle, Germany; 19 Programme area Systems Rheumatology, Deutsches Rheuma-Forschungszentrum Berlin, ein Institut der Leibniz-Gemeinschaft, Berlin, Germany; 20 German Centre for Diabetes Research (DZD), Munich, Germany

**Keywords:** Internalizing symptoms, Depression, Anxiety, Mental health, Juvenile idiopathic arthritis, Adolescents, Screening

## Abstract

**Background:**

Previous studies have shown that growing up with rheumatic conditions can fuel dissatisfaction and psychological distress, which in turn affects disease self-management and treatment adherence. Primary objective of this study was to estimate the prevalence of anxiety and depression symptoms in adolescents and young adults (AYA) with juvenile idiopathic arthritis (JIA) and to identify correlates of conspicuous screening results.

**Methods:**

Initiated as part of the COACH multicenter observational study, outpatients aged 12 to 21 years participating in the National Pediatric Rheumatological Database (NPRD) were prospectively screened for mental health using the Patient Health Questionnaire-9 (PHQ-9) and the Generalised Anxiety Disorder Scale-7 (GAD-7).

**Results:**

Data from 1,150 adolescents with JIA (mean age 15.6 ± 2.2 years; mean disease duration 7.2 ± 4.9 years, 69% female, 43% oligoarthritis, 26% polyarthritis) were analysed. Overall, 32.7% (*n* = 316) of AYA showed conspicuous screening results, of whom 30.4% reported clinically relevant suicidal or self-harm thoughts. About 19% of screened patients showed moderate to severe depressive or anxious symptoms. AYA with conspicuous screening results were older (15.8 vs. 15.2 years; *p* < 0.0001), more often female (81% vs. 64%; *p* < 0.0001) and more often overweight (25% vs. 17%; *p* = 0.006). They had higher disease activity (physician global assessment on NRS 0–10; 1.7 vs. 1.2; *p* < 0.0001), more functional limitations (CHAQ; 0.44 vs. 0.14; <0.0001) and rated their health status worse (NRS 0–10; 3.5 vs. 1.8; *p* < 0.0001) than AYA with inconspicuous screening results. Females (OR 2.33 [CI 1.53–3.56]; *p* < 0.0001), older age (OR 1.09 [CI 1.01–1.18]; *p* = 0.026), patients with more functional limitations (OR 3.36 [CI 1.98–5.72]; *p* < 0.0001), and patients with worse subjective health status (OR 1.17 [CI 1.07–1.27]; *p* < 0.0001) were more likely to have a conspicuous screening result. Regular sports participation was associated with a lower likelihood of conspicuous screening result (OR 0.69 [CI 0.49–0.98]; *p* = 0.039).

**Conclusions:**

A large-scale outpatient screening of AYA with JIA in Germany shows a high prevalence of anxiety and depression symptoms. The need for routine screening for early detection of mental health problems became apparent.

**Supplementary Information:**

The online version contains supplementary material available at 10.1186/s13075-024-03312-x.

## Introduction

Adolescence is a time of significant physical, behavioural, and psychological change [1]. Emotional, social, and mental well-being predict age-appropriate developmental trajectories, future risk for psychological impairment, and long-term health [2, 3]. In about half of mental disorders, symptoms appear well before the age of 18 [4]. For adolescents suffering from chronic disease, the common changes they experience during adolescence are compounded by the challenge of figuring out how to manage a chronic disease independently and how best to make the transition from paediatric to adult care [5]. In addition, young patients who suffer from chronic diseases show a less favourable course of mental symptoms over time [6].

Juvenile idiopathic arthritis (JIA) is the most common chronic rheumatic disease in paediatrics describing a heterogeneous group of inflammatory rheumatic diseases of unknown origin. It begins before the age of 16, persists for at least 6 weeks and is diagnosed after exclusion of any other known arthritis causes [7]. Affected individuals experience joint pain and swelling, morning stiffness, and limited mobility. Long-term consequences of JIA include joint damage, muscle weakness and atrophy, growth disturbances, and comorbid conditions like uveitis might develop during the disease course [8]. Resulting restrictions in daily life, time-consuming physical therapies, and medication side effects can fuel dissatisfaction and psychological distress, which in turn detoriates disease self-management and treatment adherence [9, 10].

While previous studies have shown that growing up with rheumatic conditions can be associated with poorer psychological functioning [11], studies on depression and anxiety in paediatric patients with JIA and the possible association of these symptoms with disease characteristics are limited and provide conflicting results [9, 12–18]. In fact, studies on mental health in JIA reported rates of depressive and anxious symptoms ranging from 7 to 36% and 7–64%, respectively, and were often limited by small sample sizes. As anxiety and depressive symptoms may be non-specific, unreported, or even concealed by patients, targeted screening methods can be used to identify mental health problems [19]. Although international professional societies and task forces advocate the routine use of psychosocial screening tools during adolescence [20–23], it has not yet been implemented in most paediatric rheumatology centres. This situation hinders early and effective provision of mental health services and might in turn impair the outcome of the underlying disease.

In this study we analysed the prevalence of anxious and depressive symptoms captured by a standardized outpatient screening in adolescents and young adults (AYA) with JIA in Germany and assessed the association of sociodemographic, clinical parameters, and patient reported outcomes of JIA with symptoms of anxiety and depression.

## Methods

### Patients

Subjects in this multicentre observational study were included within the framework of the COACH Study (Chronic Conditions in Adolescents: Implementation and Evaluation of Patient-centred Collaborative Health Care), a multi-site prospective study aiming to improve mental healthcare utilization among AYA with chronic health conditions in Germany. Using the National Paediatric Rheumatologic Database (NPRD) as established patient register, AYA from 48 paediatric rheumatology centres were actively recruited during routine consultations. This number represents approximately 75% of all centres participating in the NPRD, including the largest centres in Germany. The nationwide NPRD captures a broad spectrum of juvenile rheumatic diseases and annually collects data on disease phenomena and outcome measures using standardized physician reports and patient questionnaires. According to estimates, the number of JIA cases recorded each year corresponds to about 50% of all expected cases in Germany. Further details on this representative database, containing sociodemographic and clinical characteristics as well as treatment assignments are provided by Minden et al. [24] and Klotsche et al. [25].

Inclusion criteria for the analyses in the present study were as follows: (1) age at documentation between 12 and 21 years, (2) diagnosis of JIA according to the International League of Associations for Rheumatology (ILAR) criteria [7], and (3) fluency in the German language to complete the questionnaires. The evaluation included patients recruited between January 2019 and December 2022. The study was approved by the ethics committee of the Charité - Universitätsmedizin Berlin (EA1/044/07).

### Questionnaires on anxiety and depression symptoms

Anxiety and depressive symptoms were assessed via two standardised self-report questionnaires during routine consultations on a tablet computer (or with paper and pencil).

The Generalized Anxiety Disorder-7 (GAD-7) measuring severity of various signs of generalized anxiety disorder (GAD) over the last two weeks on a four-point scale [26]. The GAD-7 score is calculated by assigning scores of 0 to 3, to the response categories of ‘not at all’, ‘several days’, ‘more than half the days’, and ‘nearly every day’, respectively. The total score (0–21) is obtained by adding all seven questions’ scores.

To assess the presence and severity of depressive symptoms, the Patient Health Questionnaire-9 (PHQ-9) a 9-question depression scale from the Patient Health Questionnaire (PHQ) was used [27]. Analogue to the GAD-7, the questionnaire rates the frequency of symptoms on a 4-point scale (scoring 0–3) over the last two weeks, whereby overall scale scores are computed as a sum of all items (possible range 0–27).

Both instruments have high reliability and are frequently used to assess mental health problems among adolescents [28, 29].

The GAD-7 and PHQ-9 questionnaires have different cut-off recommendations. While the GAD-7 questionnaire has acceptable sensitivity and specificity with a score between 7 and 10 points [30], the optimal cut-off value according to the Youden index (maximum sum of sensitivity and specificity) for the PHQ-9 questionnaire was 7 [31]. The aim in choosing the cut-off values for the screening within our study was to detect indications of anxiety and depression at an early stage and without pathologizing unnecessarily. Therefore, we used a cut-off value of 7, which corresponds to mild to moderate symptomatology. A conspicuous screening result was defined as a score ≥ 7 in either instrument. For the analysis of associations of conspicuous screening results with sociodemographic and clinical parameters, only those patients for whom a complete PHQ-9 and GAD-7 were available were considered. When analysing the frequency and severity of depressive and anxiety symptoms separately, complete availability of the respective instrument was sufficient. Consistent with the originally defined cut-off values for PHQ-9 [32] and GAD-7 [26], we also reported the frequency of depressive and anxious symptoms according to conventional cut-off scores.

To detect the frequency of possible signs of suicidal or self-harm thoughts within the last two weeks, the final item of the PHQ-9 (item 9) was used: ‘Thoughts that you would be better off dead, or thoughts of hurting yourself in some way’. The following response categories are available: ‘never’ (score = 0), ‘on several days’ (score = 1), ‘more than half the days’ (score = 2) and ‘almost every day’ (score = 3). In case of conspicuous screening result, patients and their families received information about support services and the offer to participate in an intervention study within the framework of the COACH research network.

### Clinical data

Sociodemographic data reported by patients were age, gender and information on type of school, including lowest, middle, and highest German secondary school (University entrance qualification). Physician data on sociodemographics and anthropometrics included age, gender, height, and weight. BMI was calculated as the weight in kilograms divided by the height in metres squared. Underweight (BMI < 10th), normal weight (BMI ≥ 10th - ≤90th), overweight (BMI > 90th) and obesity (BMI > 97th) were defined according to age- and gender-specific percentiles used in the German reference system [33].

JIA-specific data collected by the physician were diagnosis, age at disease onset, disease duration, number of joints affected as well as laboratory values such as erythrocyte sedimentation rate (ESR) and C-reactive protein (CRP). Additionally, the physician assessed patient’s disease activity (physician’s global assessment, PGA) on a numerical rating scale (NRS; from 0 = no disease activity to 10 = very severe disease activity). Further, the physician recorded treatment with glucocorticoids (GCs) as well as conventional synthetic (csDMARDs) and biological (bDMARDs) disease-modifying antirheumatic drugs at the day of the documentation and within the last 12 months. Medication with systemic GCs included low-dose (< 0.2 mg/kg body weight/day) and high-dose (≥ 0.2 mg/kg body weight/day). Physician data on BMI, disease activity and number of affected joints were linked to screening results only if they were recorded in the NPRD within ≤ 3 months before or after screening.

Patient-reported data beyond GAD-7 and PHQ-9 included information on functional ability in everyday life. Data was collected using the German version of the Childhood Health Assessment Questionnaire (C-HAQ) [34]. The resulting disability index ranged from 0 to 3, whereby a value of zero indicated no functional disability and higher scores indicated light, moderate, or severe level of disability. Patients also reported the number of days they spent as inpatient due to their rheumatic disease in the past 12 months. Additionally, patients were asked to rate their general well-being, pain intensity, fatigue and coping with the rheumatic disease on a numerical rating scale ranging from 0 to 10. Based on physician- and patient-reported data, disease activity was assessed with the clinical Juvenile Arthritis Disease Activity Score in 10 joints (cJADAS-10) [35]. The cJADAS-10 considers the number of joints with active disease, physicians’ global assessment and patients’ rating on well-being. In accordance with Trincianti et al. [36], the cJADAS-10 cut-off for classification of moderate to high disease activity was 4.1 for oligoarticular JIA and 5.1 for polyarticular JIA.

The assessment of modifiable lifestyle factors included patient-reported information on physical activities in everyday life. Patients were asked, ‘On how many days of a normal week are you physically active for at least 60 minutes on a single day?’ The eight answer categories ranged from ‘On no day’ to ‘On seven days’. Based on these data it was assessed whether patients met the WHO recommended level of physical activity of 60 min daily [37]. In addition, patients were asked to rate their physical fitness on a 4-point Likert scale ranging from ‘very good’ to ‘very bad’. Patients were also asked if they exercised regularly. All lifestyle-related questions were taken from the German Health Interview and Examination Survey for Children and Adolescents (KiGGS), a long-term study conducted by the Robert Koch Institute [38].

### Statistical analysis

Categorical variables were reported by numbers and percentages, whereas continuous variables were reported by means and standard deviations (95%-confidence interval (CI)) respective medians and interquartile ranges (IQR). Chi-square tests (for categorical variables) and Mann-Whitney tests (for group comparisons) were used to estimate unadjusted differences between males and females and those with conspicuous or inconspicuous screening results. Logistic regression was used to estimate the independent contribution of each predictor on depressive and anxious symptoms. The association between clinical correlates and mental health is described by odds ratios (ORs) with 95% confidence intervals (CIs). Regression model was adjusted for date of survey. All *p*-values less than 0.05 were considered to be statistically significant. Statistical analyses were performed using IBM SPSS version 26.0 software (IBM Corp., Armonk, NY, USA).

## Results

### Patient characteristics

In total, data of 1,150 AYA with JIA could be analysed. Mean age of the study sample was 15.6 years, about two-thirds were female, while the most common JIA category was persistent oligoarthritis. Further demographic and clinical characteristics of the total study sample as well as characteristics categorized by screening result are shown in Table 1.

**Table 1:** Demographic, anthropometric, and clinical variables of the total study cohort and those stratified by screening results for anxiety and depression, unadjusted data

Variable	Total (*n* = 1.150)	Conspicuous screening result (score of ≥ 7 in either test) (*n* = 316)	Inconspicuous screening result (score of < 7 in both tests) (*n* = 651)	*P*-value
**Sociodemographic / anthropometric data**				
Age, years, mean (SD) Female gender, no. (%) BMI-SDS, mean (SD) Underweight, no. (%) Normal weight, no. (%) Overweight / obesity, no. (%)	15.6 (2.2) 790 (68.8) -0.02 (1.2) 136 (12.6) 722 (66.9) 222 (20.6)	15.8 (2.1) 255 (81.0) 0.08 (1.1) 26 (8.5) 203 (66.6) 76 (24.9)	15.2 (2.3) 415 (63.7) -0.11 (1.2) 85 (14.2) 411 (68.6) 103 (17.2)	**< 0.0001** **< 0.0001** **0.028** **0.014** 0.531 **0.006**
Type of school, no. (%)				
Lowest/middle German secondary school Highest German secondary school	382 (48.5) 405 (51.5)	98 (48.5) 104 (51.5)	224 (48.7) 236 (51.3)	0.966 0.966
**JIA specific data**				
Disease duration, years, mean (SD) Age at disease onset, years, mean (SD)	7.2 (4.9) 8.4 (4.9)	7.3 (5.1) 8.5 (5.0)	7.2 (4.8) 8.0 (4.7)	0.962 0.180
JIA category, no. (%)				
RF-positive polyarthritis RF-negative polyarthritis Systemic JIA Persistent oligoarthritis Extended oligoarthritis Psoriatic arthritis Enthesitis-related arthritis Unclassified JIA	48 (4.2) 252 (21.9) 38 (3.3) 325 (28.5) 171 (14.9) 87 (7.6) 174 (15.1) 41 (3.6)	17 (5.4) 82 (25.9) 10 (3.2) 70 (22.2) 54 (17.1) 27 (8.5) 47 (14.9) 7 (2.2)	22 (3.4) 141 (21.7) 21 (3,2) 191 (29.3) 99 (15.2) 50 (7.7) 93 (14.3) 29 (4.5)	0.139 0.140 0.957 **0.017** 0.457 0.646 0.814 0.084
cJADAS-10, (0–30), mean (SD) PGA score, NRS 0–10, mean (SD) Inactive disease*, no. (%) No. of joints with active disease, mean (SD)	4.6 (4.7) 1.4 (2.0) 480 (53.6) 0.9 (2.5)	6.1 (5.1) 1.7 (2.1) 114 (46.9) 1.1 (2.8)	3.8 (4.3) 1.2 (1.8) 294 (57.8) 0.8 (2.5)	**< 0.0001** **< 0.0001** **0.005** 0.096
**Laboratory parameters**, median (Interquartile Range)				
C-reactive protein, mg/l Erythrocyte sedimentation rate, mm/h	0.8 (3.2) 7.0 (10.0)	0.9 (3.7) 7.0 (11.3)	0.6 (2.5) 6.0 (8.0)	**0.022** **0.007**
**Drug therapy (past 12 months)**				
Low-dose GCs (< 0.2 mg/kg/day), no. (%) High-dose GCs (≥ 0.2 mg/kg/day), no. (%) Any conventional synthetic DMARD, no. (%) Any biologic DMARD, no. (%)	72 (6.8) 38 (3.6) 458 (40.9) 433 (38.5)	26 (8.8) 16 (5.5) 137 (44.8) 128 (41.0)	30 (5.0) 14 (2.4) 248 (39.0) 240 (37.7)	**0.028** **0.016** 0.091 0.320
**Patient-reported data**				
C-HAQ total score (0–3), mean (SD) Well-being, NRS 0–10, mean (SD) Pain intensity, NRS 0–10, mean (SD) Fatigue, NRS 0–10, mean (SD) Coping, NRS 0–10, mean (SD) Hospital days (past 12 month), mean (SD)	0.24 (0.5) 2.5 (2.5) 2.4 (2.7) 2.2 (2.9) 1.7 (2.3) 2.1 (8.5)	0.44 (0.6) 3.5 (2.6) 3.5 (2.9) 4.1 (3.3) 2.8 (2.6) 2.4 (6.5)	0.14 (0.3) 1.8 (2.1) 1.9 (2.5) 1.2 (2.1) 1.1 (1.8) 1.7 (6.0)	**< 0.0001** **< 0.0001** **< 0.0001** **< 0.0001** **< 0.0001** 0.073
**Modifiable lifestyle factors**				
Physical fitness^€^, no. (%) Regular sports participation, no. (%) Sufficient physical activity^¥^, no. (%)	554 (62.1) 599 (63.9) 192 (18.3)	120 (46.5) 154 (54.4) 47 (16.6)	398 (70.1) 401 (68.1) 112 (18.9)	**< 0.0001** **< 0.0001** 0.407

JIA, juvenile idiopathic arthritis; RF, rheumatoid factor; cJADAS-10, 10-joint clinical Juvenile Arthritis Disease Activity Score; PGA, physician’s global assessment; C-HAQ, Childhood Health Assessment Questionnaire; GC, glucocorticoid; DMARD, disease-modifying antirheumatic drug; NRS, Numerical Rating Scale. *Defined by a PGA score of zero. ^**€**^Defined as ‘good’ or ‘very good’ self-reported on a five-point Likert scale. ^¥^self-reported physical activity of at least 60 min/day (WHO recommendation)

### Screening results

Based on 1,150 screenings, 1,030 complete GAD-7s and 984 complete PHQ-9s could be considered. A complete PHQ-9 and GAD-7 was available for 967 patients.

Overall, 32.7% of AYA (*n* = 316) had a conspicuous screening result (score of ≥ 7 on either screening tool), with a prevalence of 38.1% (*n* = 255) among females and 20.3% (*n* = 60) among males (*p* < 0.0001) (Results not shown). While 41.9% (*n* = 62) in the age group 18–21 years had a conspicuous screening result, the proportion among 15-17-year olds was 35.0% (*n* = 164) and among 12-14-year olds 26.0% (*n* = 87, *p* = 0.001). Patients with RF + polyarthritis (43.6%) and RF- polyarthritis (36.8%) most frequently showed a conspicuous screening result (score of ≥ 7 in either instrument), followed by patients with extended oligoarthritis (35.3%), psoriatic arthritis (35.1%), enthesitis-associated arthritis (33.6%), systemic JIA (32.3%), and persistent oligoarthritis (26.8%).

Based on complete GAD-7 (*n* = 1,030) and PHQ-9 (*n* = 984), 26.2% reported mild to moderate symptoms (score ≥ 7) of anxiety and 25.8% of depression. Details on screening results for depressive and anxiety symptoms stratified by gender and age group for different cut-offs are presented in Table 2 (Suppl. Table S1, S2, S3).

**Table 2:** Screening results for depressive (PHQ-9) and anxiety (GAD-7) symptoms stratified by gender and age group for different cut-offs

Variables	PHQ-9, mean (SD)	PHQ-9 score ≥ 7 no (%)	PHQ-9 score ≥ 10 no (%)	GAD-7, mean (SD)	GAD-7 score ≥ 7 no (%)	GAD-7 score ≥ 10 no (%)
**total**	4.6 (4.6)	270 (26.2)	147 (14.9)	4.4 (4.9)	254 (25.8)	146 (14.2)
12–14 years	3.7 (4.3)	65 (19.3)	37 (11.0)	3.6 (3.9)	69 (19.5)	31 (8.8)
15–17 years	4.8 (5.0)	125 (26.6)	71 (15.1)	4.4 (4.6)	138 (27.8)	73 (14.7)
18–21 years	6.0 (5.7)	52 (34.4)	36 (23.8)	5.8 (5.5)	61 (37.7)	40 (24.7)
**female**	5.3 (5.3)	204 (30.2)	127 (18.8)	4.9 (4.7)	219 (30.7)	119 (16.7)
12–14 years	4.2 (4.7)	52 (22.9)	33 (14.5)	3.9 (4.0)	51 (21.4)	22 (9.2)
15–17 years	5.6 (5.3)	104 (32.4)	62 (19.3)	5.1 (4.7)	114 (33.5)	62 (18.2)
18–21 years	6.8 (5.9)	47 (39.5)	32 (26.9)	6.3 (5.5)	53 (41.7)	34 (26.8)
**male**	3.0 (3.7)	40 (13.4)	17 (5.7)	3.1 (3.9)	50 (15.9)	26 (8.3)
12–14 years	2.6 (2.8)	12 (11.0)	3 (2.8)	3.0 (3.6)	18 (15.8)	9 (7.9)
15–17 years	3.3 (4.1)	21 (14.1)	9 (6.0)	3.0 (4.0)	24 (15.3)	11 (7.0)
18–21 years	3.25 (4.0)	5 (15.6)	4 (12.5)	3.9 (4.8)	7 (20.6)	5 (14.7)

PHQ-9 (score 0–27), Patient Health Questionnaire-9; GAD-7 (score 0–21), Generalized Anxiety Disorder Scale-7.

In patients with polyarticular disease, both moderate to severe depressive (19.5%) and anxiety symptoms (16.8%) were registered more frequently than in patients with oligoarthritis (14.8% resp. 13.2%).

Among all screened patients, 15.1% were receiving psychotherapeutic (12.2%) or psychopharmacological (6.3%) treatment. Among patients with conspicuous screening result, 32.1% were undergoing psychotherapeutic (25.9%) or psychopharmacological (13.6%) treatment.

**Fig. 1:**
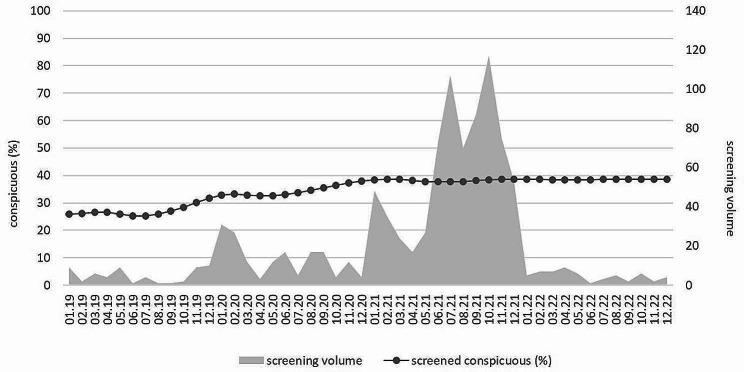
Monthly screenings and percent conspicuous rate

The number of screenings and monthly positive rates during the observation period are shown in Fig. 1.

### Suicidal or self-harm thoughts

A total of 1027 patients responded to the question on signs of suicidal or self-harm thoughts (PHQ-9 item 9), of which 12.2% (*n* = 125) answered affirmatively (Table 3). The majority (*n* = 90) selected “on several days” (score = 1) as their answer. While 14.9% of girls reported having such thoughts on at least several days, the proportion among boys was 5.8% (p = < 0.0001). Different frequencies were registered among 12-14-year olds (9.7%, *n* = 34), 15-17-year olds (12.1%, *n* = 60), and 18-21-year olds (19.0%, *n* = 31).

Among all patients with conspicuous screening result (*n* = 316), 30.4% reported signs of suicidal or self-harm thoughts, with girls (33.7%, *n* = 86) stating such thoughts significantly more often (*p* = 0.004) than boys (15.0%, *n* = 10).

Signs of suicidal or self-harm thoughts were registered most frequently in the age group 18–21 years (37.1%, *n* = 23), followed by the age group 12–14 (34.5%, *n* = 30) and 15–17 years (25.6%, *n* = 43). Patient characteristics according to the question on suicidal and self-harm thoughts are shown in Table 3.

**Table 3:** Demographic, anthropometric, and clinical variables stratified by signs of self-harm or suicidal ideations, unadjusted data

Variable	Conspicuous screening result (score of PHQ-9 item 9 ≥ 1) (*n* = 125)	Inconspicuous screening result (score of PHQ-9 item 9 = 0) (*n* = 901)	*p*-value
**Sociodemographic / anthropometric data**			
Age, years, mean (SD) Female, no. (%) Normal weight, no. (%) Overweight, no. (%)	15.9 (2.1) 106 (84.8) 69 (58.5) 32 (27.1)	15.3 (2.2) 607 (67.4) 580 (68.6) 161 (19.1)	**0.005** **< 0.0001** **0.027** **0.040**
**JIA specific data**			
Disease duration, years, mean (SD) Age at disease onset, years, mean (SD)	8.3 (5.1) 7.7 (5.0)	7.1 (4.9) 8.3 (4.7)	**0.026** 0.208
JIA category, no. (%)			
RF-positive polyarthritis RF-negative polyarthritis Systemic JIA Persistent oligoarthritis Extended oligoarthritis Psoriatic arthritis Enthesitis-related arthritis	8 (6.3) 32 (25.4) 1 (0.8) 34 (27.0) 27 (21.4) 8 (6.3) 11 (8.7)	34 (3.8) 203 (22.5) 32 (3.6) 245 (27.2) 140 (15.5) 70 (7.8) 137 (15.2)	0.170 0.468 0.100 0.967 0.092 0.575 0.053
cJADAS-10, 0–30, mean (SD) PGA score, NRS 0–10, mean (SD) No. of joints with active disease, mean (SD)	5.3 (4.7) 1.5 (2.1) 0.69 (2.4)	4.5 (4.7) 1.4 (1.9) 0.92 (2.6)	**0.020** 0.683 **0.036**
**Drug therapy (past 12 months)**			
Systemic GCs, no. (%) Any conventional synthetic DMARD, no. (%) Any biologic DMARD, no. (%)	14 (11.8) 56 (45.5) 46 (37.1)	68 (8.2) 356 (40.6) 347 (39.3)	0.196 0.298 0.638
**Patient-reported data**			
C-HAQ total score, mean (SD) Well-being, NRS 0–10, mean (SD) Pain intensity, NRS 0–10, mean (SD) Fatigue, NRS 0–10, mean (SD) Coping, NRS 0–10, mean (SD)	0.38 (0.6) 3.3 (2.6) 3.0 (2.8) 4.1 (3.5) 2.5 (2.5)	0.22 (0.4) 2.3 (2.4) 2.3 (2.7) 1.9 (2.7) 1.5 (2.2)	**0.004** **< 0.0001** **0.006** **< 0.0001** **< 0.0001**

JIA, juvenile idiopathic arthritis; RF, rheumatoid factor; cJADAS-10, 10-joint clinical Juvenile Arthritis Disease Activity Score; PGA, physician’s global assessment; C-HAQ, Childhood Health Assessment Questionnaire; GC, glucocorticoid; DMARD, disease-modifying antirheumatic drug; NRS, Numerical Rating Scale; PHQ-9, Patient Health Questionnaire-9. The frequency of possible signs of suicidal or self-harm thoughts are based on PHQ-9 item 9: ‘Thoughts that you would be better off dead, or thoughts of hurting yourself in some way’ with response categories ranging from ‘never’ (score = 0), ‘on several days’ (score = 1), ‘more than half the days’ (score = 2) and ‘almost every day’ (score = 3). The association of sociodemographic and clinical parameters with signs of suicidal or self-harm thoughts was investigated using logistic regression analysis.

### Factors associated with conspicuous screening result

**Fig. 2:**
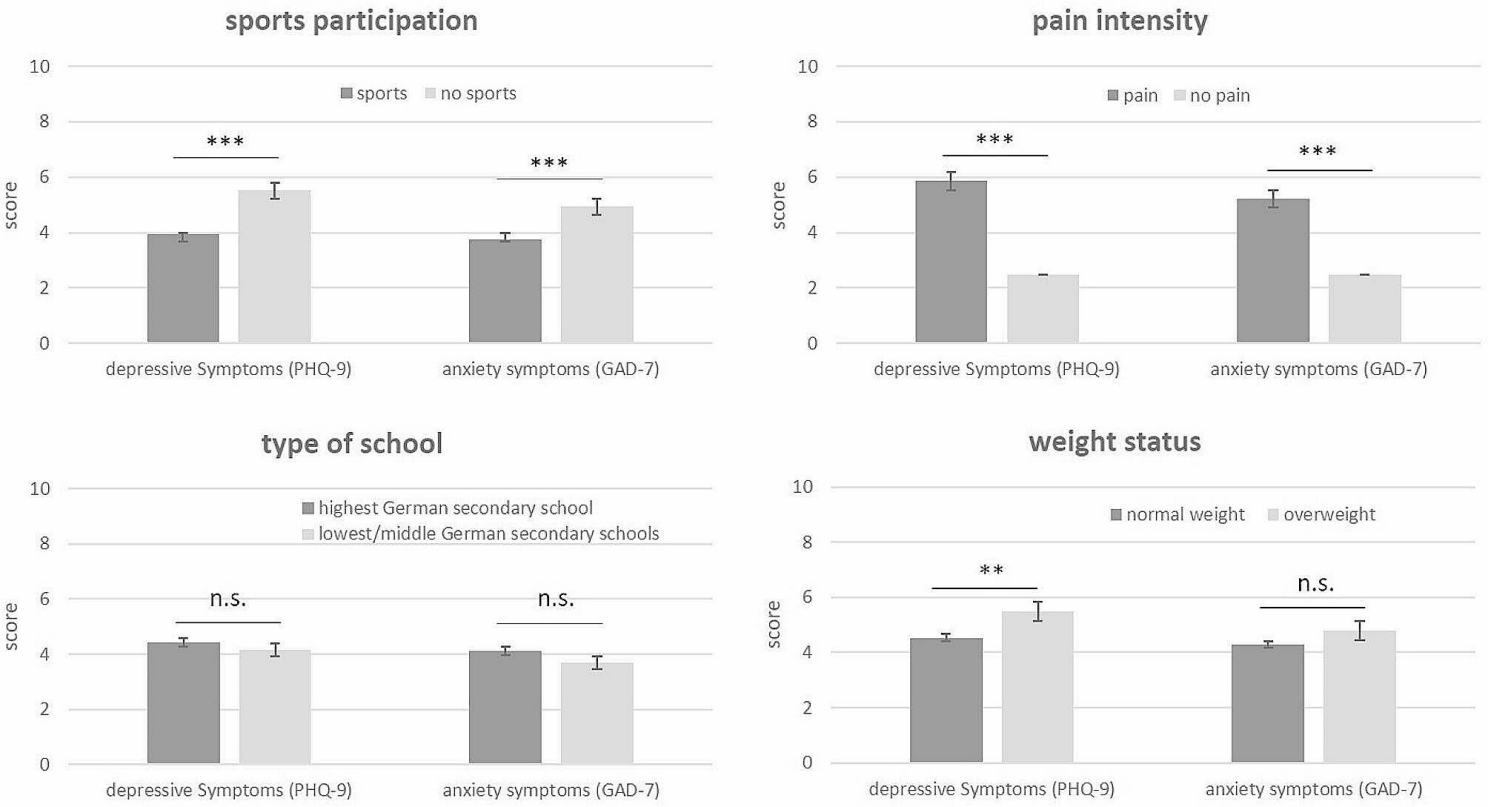
Differences in adjusted mean PHQ-9 and GAD-7 scores by sports participation, pain, type of school, and weight status. Legend: Bars represent adjusted means and 95% CI. GAD-7 (score 0–21), Generalized Anxiety Disorder Scale-7; PHQ-9 (score 0–27), Patient Health Questionnaire-9. Group differences were tested by Mann-Whitney test. ***p < 0.0001; **p < 0.01; *p < 0.05

AYA with conspicuous screening result were more often female (81% vs. 64%; *p* < 0.0001) than male, older (15.8 vs. 15.2 years; *p* < 0.0001), and more often overweight (25% vs. 17%; *p* = 0.006) than those with inconspicuous screening (Table 1). Type of school did not differ between patients with conspicuous and inconspicuous screening result, which is also reflected in the mean scores of GAD-7 and PHQ-9 (Fig. 2). AYA with conspicuous screening result reported a higher disease activity and more severe functional limitations. Further detailed information on sociodemographic and clinical parameters, including patient-reported outcomes associated with conspicuous screening results, are presented in Table 1.

Patients who received conventional synthetic DMARDs reported moderate to severe (score ≥ 10) depressive symptoms more frequently than patients who were not treated with conventional synthetic DMARDs (*p* = 0.029) (Results not shown). However, there were no significant differences in the frequency of moderate to severe anxiety symptoms (*p* = 0.121) or between patients treated and not treated with biologics. Regular sports participants were less likely to have a conspicuous screening result. Figure 2 shows that patients who do not exercise regularly and suffer from pain displayed higher mean screening scores for depression and anxiety symptoms than patients without pain and with regular exercise.

Increasing age, female gender, more severe functional disability, and worse well-being were independently associated with a higher likelihood of a conspicuous screening result in multivariable analysis (Table 4).

**Table 4:** Association of internalizing symptoms with clinical and sociodemographic parameters, adjusted OR and 95% CI

	aOR (adjusted for date of survey)	*p*
**Sociodemographic** / **anthropometric data**		
Age	1.09 (1.01–1.18)	**0.026**
Female gender	2.33 (1.53–3.56)	**< 0.0001**
Overweight > 90. Percentile _vs. ≤90 p_	1.39 (0.92–2.09)	0.120
**JIA specific data**		
PGA score*	0.96 (0.87–1.05)	0.385
RF-positive polyarthritis RF-negative polyarthritis Systemic JIA Persistent oligoarthritis Extended oligoarthritis Psoriatic arthritis Enthesitis-related arthritis	2.31 (0.59–9.11) 2.30 (0.72–7.45) 3.76 (0.91–15.47) 2.13 (0.66–6.79) 2.30 (0.70–7.54) 2.22 (0.64–7.76) 3.01 (0.91–9.93)	0.231 0.161 0.066 0.203 0.169 0.211 0.070
**Patient-reported data**		
Functional disability (C-HAQ)*	3.36 (1.98–5.72)	**< 0.0001**
Well-being*	1.17 (1.07–1.27)	**< 0.0001**
**Lifestyle factors**		
Regular sports participation	0.69 (0.49–0.98)	**0.039**

JIA, juvenile idiopathic arthritis; RF, rheumatoid factor; cJADAS-10, 10-joint clinical Juvenile Arthritis Disease Activity Score; C-HAQ, Childhood Health Assessment Questionnaire. Internalizing symptoms defined as score of ≥ 7 in at least one of two screening instruments (PHQ-7/GAD-7). GAD-7 (score 0–21), Generalized Anxiety Disorder Scale-7; PHQ-9 (score 0–27), Patient Health Questionnaire-9. A logistic regression model was performed. *higher scores indicate worse PGA, functional disability, and well-being.

## Discussion

Based on one of the most extensive screening analyses of internalizing symptoms conducted in paediatric JIA patients to date, we found a high prevalence of primary mild depressive or anxious symptoms. While the frequency of anxious symptoms was similar to that of depressive symptoms, more than one in ten patients reported signs of suicidal or self-harm thoughts.

Few previous studies on internalizing symptoms in JIA have highlighted an increased risk in specific outcomes such as depression and anxiety compared to the general population [9, 39–41]. Methodological discrepancies, but also sometimes small heterogeneous samples, differences in disease durations/activities, and the high rate of mental health problems in the general population may explain why some previous studies have stated no increased risk of mental health problems in JIA patients compared to general population controls [13, 15–18, 43]. As our study did not include a control group, an exact comparison with the general population is not possible. However, the prevalence of anxiety symptoms found in our study seems to be slightly higher than German normative data [44] and also slightly higher than the prevalence recently reported among adolescents from the general population [45]. A previous study from the German general population on prevalence and severity of depressive symptoms showed results comparable to those found in our patients from the same age group [46]. Another study conducted among adolescents from the German general population, however, reported a higher mean PHQ-9 score compared to our findings [47]. In these studies, and also in ours, screening took place immediately before and/or during the Corona pandemic, a period in which the school-aged population was exposed to increased psychological stress [48]. However, potential negative effects were neither reflected in the severity of depressive symptoms (PHQ-9) in an adolescent sample from the German general population [47] nor in the frequency of psychological diagnoses in adolescents with JIA [49], at least until 2021.

In our study, a high proportion of patients showed signs of suicidal or self-harm thoughts, which were based on the final item of PHQ-9. Although it is widely used as a single scale for studies on the prevalence of suicidal ideas, however, the predictive value of PHQ-9 item 9 on suicide risk remains unclear and the combination with self-harm in one item can be misleading [50]. Alternatively, one could add a more in-depth evidence-based self-report for the assessment of suicidal ideas and suicidality in case of PHQ-9 item 9 > score 0. This might improve acceptance of such an assessment amongst clinicians. Moreover, research highlights the fact, that PHQ-9 item-9 might not only result in a very high false-positive rate, but also lack specificity, strengthening the call for a more reliable and valid procedure that yet need to be further detailed and examined [51]. Defined clinical pathways to implement screening programs might thereby aid with an efficient detecting and managing of patients who are at risk for suicide [52]. National and international organizations have issued guidelines for the prevention of suicide in young people including comprehensive diagnostics [53].

Consistent with trends in the general population [42] and those previously reported in JIA [14, 18, 41, 54], our results showed that females were more likely to have conspicuous screening results and higher symptom severity than males. Moreover, we have shown that the risk for conspicuous depressive and anxiety symptoms increases significantly with age, with older patients more frequently reporting higher symptom severity. Thus, our results confirm previous studies on the association between age and mental health in JIA [14, 17, 19]. As clinically significant symptoms occurred most frequently in early adulthood, it probably negatively affects the already challenging transition process to internal medicine rheumatology.

A previous work by Kyllönen et al. [54] focused on the impact of the age at JIA onset on behavioural disorders and summarized that this may have implications for future mental health. In our study, we found no association between age at JIA onset and internalizing symptoms, however, due to our cross-sectional study design, comparison with this previous study remains difficult.

Our results do not confirm previous findings by El-Najjar et al. [55], demonstrating that longer disease duration is associated with higher risk of depressive symptoms. Instead, the comparatively long disease duration of our patients of more than 7 years supports the theory that longer disease duration allows some adolescents to adapt and improve. This assumption corresponds with the hypothesis of Butler et al. [56], who assumed that symptoms would improve after initial adjustment to disease, and found a slight decrease in symptoms at six-month follow-up. This is also supported by an earlier study showing that adolescents with lower anxiety and depressive symptoms tended to have a longer disease duration [57].

Our results support previous findings, showing that patients with a polyarticular course are at higher risk for depression than patients from other categories [9, 14]. In contrast, El-Najjar et al. [55] and Fair et al. [17] did not find a statistically significant difference in symptom severity between various JIA categories. This discrepancy might be attributed to small sample sizes for some categories.

Our findings are consistent with a number of previous studies reporting associations between markers of disease activity and internalizing symptoms such as depression and anxiety [13, 15, 55, 58]. However, Ding et al. [16] and Fair et al. [17] did not find any association between disease activity and psychological functioning, which may be due to the fact that most of patients studied had either inactive or low disease activity. Similar to Tarakci et al. [13] and Roemer et al. [58], but in contrast to few previous studies [14, 55] we did not find an association between the number of active joints and conspicuous screening results. Hanns et al. [14] noted that a higher active joint count correlated with higher depressive scores in the first year, but this relationship was no longer significant after follow up four years later. A possible explanation for the conflicting baseline result reported by Hanns et al. [14] is the comparatively very low number of active joints in our study sample.

It should be noted that all of these previous studies have used different assessment tools and are from mixed populations of children and adolescents, both of which may be factors contributing to differences in findings.

Another important aspect is that due to the cross-sectional nature of our and most previous studies, the directionality of the associations remains unknown. However, it seems plausible that depressive symptoms may lead to worsening disease activity, especially when patients do not adhere to medical care because of their psychological symptoms. In other disease processes, including childhood lupus, depressive symptoms have already been associated with poor treatment adherence [10], highlighting the importance of mental health screening.

We observed that patients receiving conventional synthetic DMARD therapy were more likely to experience moderate to severe depressive symptoms than those without such therapy. One explanation might include known side effects such as nausea and vomiting, particularly caused by methotrexate [59], which can negatively affect mood and quality of life.

According to results of our study, patients with conspicuous screening results rated their physical functioning as measured by CHAQ significantly worse than those with inconspicuous screening results. Furthermore, a higher CHAQ sum score significantly increased the likelihood of a conspicuous screening result in multivariable analyses. These results are thus in line with a number of previous studies [9, 13, 14, 16, 17, 55]. Moreover, Hanns et al. [14] even showed that a higher baseline depressive score predicted greater functional impairment and pain 1 to 4 years later.

As in most previous studies [14, 17, 55, 57, 58], patients with more severe pain were more likely to report symptoms of depression and anxiety. It is well known that pain is a risk factor for the development of mental disorders, especially in other painful conditions such as fibromyalgia [60]. Intervention studies focusing on coping strategies for pain may help improve mental health outcomes in the future. Similar to that observed for pain, patients with conspicuous screening result reported significantly worse coping with their rheumatic disease, more severe fatigue, and poorer patient-reported global assessment of current health compared to those with inconspicuous screening result. Some patient-reported outcomes were associated with depressive or anxious symptoms independently of objective parameters such as the number of active joints, suggesting that psychological comorbidity may develop independently of somatic disease activity. Consequently, the risk for depression and anxiety should not be derived only from the objectifiable burden of disease, which again highlights the need for regular, no-cause screening for depressive and anxiety symptoms in JIA.

This is the first study examining and demonstrating a significant association between participation in sports and mental health outcomes in adolescents with JIA. This was already shown in an earlier study among healthy adolescents [61]. However, data on the impact of regular exercise on mental health in adolescents with JIA are still lacking and should be part of future research.

### Strength and limitations

The strengths of our study include a multicentre outpatient screening based on one of the most extensive JIA cohorts to date. With the integration of screening into an existing large patient register and a JIA category distribution that differs only slightly from that previously reported for Europe [62], we can assume a representative sample. This study provides new insights on prevalence and severity of depression and anxiety symptoms, taking into account sociodemographic, clinical, and personal factors, as well as general and disease-specific tools.

Nevertheless, our results must be interpreted in light of several limitations. Due to the cross-sectional nature of this study, courses of mental health issues could not be analysed and causation cannot be assessed. In order to detect symptoms of anxiety and depression at an early stage, we chose a cut-off value of 7 in the present study. GAD-7 and PHQ-9 are sensitive, self-administered screening instruments for mental disorders that are typically used in outpatient and primary care settings for referral to mental health specialists. However, they cannot be used as a replacement for clinical assessment, and additional evaluations should be performed to confirm a diagnosis of a mental disorder. At the beginning of our screening phase, no PHQ-9 version validated for individuals aged < 18 existed. We therefore consistently used the version for individuals aged ≥ 18 years.

Although we were able to consider many associations with various variables, we do not have information on all potential risk factors and behaviours, such as family characteristics, socioeconomic status, education level, diet, or other stressful life events. Furthermore, we are not able to provide information about the countries of origin, but assume that they were mainly in Europe. Due to screening within an existing patient register, the results are based exclusively on data from patients receiving paediatric rheumatology care.

As the questionnaire was only offered in German, the migration share among the study participants is probably lower than in the general German population. Although we adjusted for survey date in our analyses, we cannot rule out pandemic-related influences that may have contributed to a temporary effect on individual mental well-being.

Mental health screening among adolescents and young adults with JIA conducted in routine outpatient care indicates a similarly high prevalence of anxiety and depression symptoms as in the general population. However, these young people already have to manage their somatic chronic disease and the psychological comorbidities can impede the management of the chronic disease. Therefore, the need for routine screening to detect mental health problems has become apparent.

### Electronic supplementary material

Below is the link to the electronic supplementary material.

Supplementary Material 1

## Data Availability

The datasets used and/or analysed during the current study are available from the corresponding author on reasonable request.

## Abbreviations

AYA: adolescents and young adults
BMI: Body Mass Index
CHAQ: Childhood Health Assessment Questionnaire
cJADAS-10: clinical Juvenile Arthritis Disease Activity Score in 10 joints
COACH: Chronic Conditions in Adolescents: Implementation and Evaluation of Patient-centered Collaborative Health Care
CRP: C-reactive protein
bDMARDs: biological disease-modifying antirheumatic drugs
csDMARDs: conventional synthetic disease-modifying antirheumatic drugs
ESR: Erythrocyte sedimentation rate
GAD-7: Generalised Anxiety Disorder Scale-7
GCs: Glucocorticoids
ILAR: International League of Associations for Rheumatology
JIA: juvenile idiopathic arthritis
NPRD: National Paediatric Rheumatological Database
NRS: Numerical Rating Scale
PGA: Physician’s global assessment
PHQ-9: Patient Health Questionnaire-9
