# Supplementary material for: Anxiety and depression symptoms in adolescents and young adults with juvenile idiopathic arthritis: results of an outpatient screening
Source: Arthritis Res Ther. 2024 Apr 10;26:82. doi: 10.1186/s13075-024-03312-x (PMC11005270; doi:10.1186/s13075-024-03312-x)
Supplement: Supplementary file 1 — Supplementary Material 1 [file 13075_2024_3312_MOESM1_ESM.docx]

**Table S1.** Questionnaire results stratified by gender

| **Questionnaire (scale)** | **Females** | | **Males** | | **P-value** |
| --- | --- | --- | --- | --- | --- |
|  | Mean score ± SD (N) | Median score (IQR) | Mean score ± SD (N) | Median score (IQR) |  |
| ***GAD-7*** |  |  |  |  |  |
| Feeling nervous, anxious, or on edge (0-3) | 0.8 ± 0.9 (774) | 1 (0; 1) | 0.5 ± 0.8 (337) | 0 (0; 1) | <0.0001 |
| Inability to stop or control worries (0-3) | 0.6 ± 0.8 (752) | 0 (0; 1) | 0.3 ± 0.6 (332) | 0 (0; 0) | <0.0001 |
| Excessive worries about different things (0-3) | 0.8 ± 0.9 (765) | 0.5 (0; 1) | 0.5 ± 0.7 (333) | 0 (0; 1) | <0.0001 |
| Trouble relaxing (0-3) | 0.8 ± 0.9 (755) | 1 (0; 1) | 0.6 ± 0.8 (332) | 0 (0; 1) | <0.0001 |
| Being so restless that it's hard to sit still (0-3) | 0.5 ± 0.8 (735) | 0 (0; 1) | 0.4 ± 0.8 (333) | 0 (0; 1) | 0.014 |
| Becoming easily annoyed or irritable (0-3) | 1.0 ± 0.9 (760) | 1 (0; 1) | 0.7 ± 0.8 (337) | 0 (0; 1) | <0.0001 |
| Feeling afraid as if something awful might happen (0-3) | 0.5 ± 0.8 (744) | 0 (0; 1) | 0.3 ± 0.7 (327) | 0 (0; 0) | <0.0001 |
| *GAD7 – sum score* (0-21) | 4.9 ± 4.8 (714) | 4 (1; 7) | 3.1 ± 3.9 (314) | 2 (0; 5) | <0.0001 |
| ***PHQ-9*** |  |  |  |  |  |
| Little interest or pleasure in doing things (0-3) | 0.7 ± 0.8 (753) | 0 (0; 1) | 0.5 ± 0.7 (333) | 0 (0; 1) | <0.0001 |
| Feeling down, depressed, or hopeless (0-3) | 0.6 ± 0.8 (747) | 0 (0; 1) | 0.3 ± 0.6 (324) | 0 (0; 0) | <0.0001 |
| Trouble falling or staying asleep, or sleeping too much (0-3) | 0.9 ± 1.0 (757) | 1 (0; 1) | 0.5 ± 0.8 (333) | 0 (0; 1) | <0.0001 |
| Tiredness or the feeling of having little energy (0-3) | 1.1 ± 1.0 (769) | 1 (0; 2) | 0.7 ± 0.8 (340) | 1 (0; 1) | <0.0001 |
| Poor appetite or overeating (0-3) | 0.8 ± 0.9 (748) | 0 (0; 1) | 0.5 ± 0.7 (330) | 0 (0; 1) | <0.0001 |
| Feeling bad about yourself, or that you are a failure or have let yourself or your family down (0-3) | 0.6 ± 0.9 (744) | 0 (0; 1) | 0.3 ± 0.6 (323) | 0 (0; 0) | <0.0001 |
| Trouble concentrating on things (0-3) | 0.6 ± 0.8 (754) | 0 (0; 1) | 0.4 ± 0.7 (330) | 0 (0; 1) | 0.003 |
| Moving or speaking so slowly that other people could have noticed? Or so fidgety or restless that you have been moving a lot more than usual (0-3) | 0.3 ± 0.6 (720) | 0 (0; 0) | 0.2 ± 0.6 (321) | 0 (0; 0) | <0.0001 |
| Thoughts that you would be better off dead, or thoughts of hurting yourself in some way (0-3) | 0.2 ± 0.6 (713) | 0 (0; 0) | 0.1 ± 0.3 (312) | 0 (0; 0) | <0.0001 |
| *PHQ-9 – sum score (0-27)* | 5.3 ± 5.3 (683) | 4 (1; 8) | 3.0 ± 3.7 (300) | 2 (0; 4) | <0.0001 |

GAD-7, Generalized Anxiety Disorder-7; PHQ-9, Patient Health Questionnaire-9; SD, standard deviation; IQR, Interquartile range.

|  | **Depression** (PHQ-9, n = 984, female: 683 [69.4%]) | | | |  |
| --- | --- | --- | --- | --- | --- |
|  | | | | | |
| **Variables** | **none / minimal**  **(score 0-4)** | **mild**  **(score 5-9)** | **moderate**  **(score 10-14)** | **moderately severe**  **(score 15-19)** | **severe**  **(score 20-27)** |
| **N (%)** | 604 (61.4) | 226 (23.0) | 97 (9.9) | 42 (4.3) | 14 (1.4) |
| **Female,** no. (%) | 378 (55.3) | 170 (24.9) | 83 (12.2) | 40 (5.9) | 12 (1.8) |
| 12-14 years | 145 (63.6) | 49 (1.5) | 23 (10.1) | 9 (3.9) | 2 (0.9) |
| 15-17 years | 171 (52.5) | 88 (27.0) | 44 (13.5) | 16 (4.9) | 7 (2.1) |
| 18-21 years | 55 (45.5) | 32 (26.4) | 16 (13.2) | 15 (12.4) | 3 (2.5) |
| **Male,** no. (%) | 226 (75.3) | 56 (18.7) | 14 (4.7) | 2 (0.7) | 2 (0.7) |
| 12-14 years | 87 (79.8) | 19 (17.4) | 3 (2.8) | 0 (0.0) | 0 (0.0) |
| 15-17 years | 107 (71.3) | 33 (22.0) | 7 (4.7) | 1 (0.7) | 2 (1.3) |
| 18-21 years | 25 (78.1) | 3 (9.4) | 3 (9.4) | 1 (3.1) | 0 (0.0) |
|  | | | | |  |
|  | **Anxiety** (GAD-7, n = 1030, female: 714 [69.4%]) | | | |  |
|  | | | | | |
| **Variables** | **none / minimal**  **(score 0-4)** | **mild**  **(score 5-9)** | **moderate**  **(score 10-14)** | **severe**  **(score 15-21)** |  |
| **N (%)** | 636 (61.8) | 247 (24.0) | 99 (9.6) | 47 (4.6) |  |
| **Female,** no. (%) | 401 (56.2) | 194 (27.2) | 78 (10.9) | 41 (5.7) |  |
| 12-14 years | 150 (63.0) | 66 (27.7) | 17 (7.1) | 5 (2.1) |  |
| 15-17 years | 184 (54.1) | 94 (27.6) | 41 (12.1) | 21 (6.2) |  |
| 18-21 years | 61 (48.0) | 32 (25.2) | 20 (15.7) | 14 (11.0) |  |
| **Male,** no. (%) | 235 (74.8) | 53 (16.9) | 20 (6.4) | 6 (1.9) |  |
| 12-14 years | 86 (75.4) | 19 (16.7) | 8 (7.0) | 1 (0.9) |  |
| 15-17 years | 118 (75.2) | 28 (17.8) | 7 (4.5) | 4 (2.5) |  |
| 18-21 years | 23 (67.6) | 6 (17.6) | 4 (11.8) | 1 (2.9) |  |

**Table S2.** Patients with different levels of internalizing symptoms by gender and age (conventional cut-offs)

Legend: PHQ-9 (score 0-27), Patient Health Questionnaire-9; GAD-7 (score 0-21), Generalized Anxiety Disorder Scale-7.

|  | **Depression** (PHQ-9, n = 983, female: 683 [69.4%]) | | | |  |
| --- | --- | --- | --- | --- | --- |
|  | | | | | |
| **Variables** | **none / minimal**  **(score 0-4)** | **mild**  **(score 5-9)** | **moderate**  **(score 10-14)** | **moderately severe**  **(score 15-19)** | **severe**  **(score 20-27)** |
| **N (%)** | 604 (61.4) | 226 (23.0) | 97 (9.9) | 42 (4.3) | 14 (1.4) |
| **JIA category,** no. (%) |  |  |  |  |  |
| RF-positive polyarthritis  RF-negative polyarthritis  Systemic JIA  Persistent oligoarthritis  Extended oligoarthritis  Psoriatic arthritis  Enthesitis-related arthritis | 20 (51.3)  141 (62.1)  21 (65.6)  176 (66.2)  93 (58.9)  40 (51.9)  84 (59.2) | 9 (23.1)  44 (19.4)  6 (18.8)  54 (20.3)  38 (24.1)  24 (31.2)  42 (29.6) | 8 (20.5)  24 (10.6)  4 (12.5)  26 (9.8)  16 (10.1)  10 (13.0)  8 (5.6) | 2 (5.1)  15 (6.6)  1 (3.1)  6 (2.3)  8 (5.1)  1 (1.3)  7 (4.9) | 0 (0)  3 (1.3)  0 (0)  4 (1.5)  3 (1.9)  2 (2.6)  1 (0.7) |
|  | | | | |  |
|  | **Anxiety** (GAD-7, n = 1029, female: 714 [69.4%]) | | | |  |
|  | | | | | |
| **Variables** | **none / minimal**  **(score 0-4)** | **mild**  **(score 5-9)** | **moderate**  **(score 10-14)** | **severe**  **(score 15-21)** |  |
| **N (%)** | 636 (61.8) | 247 (24.0) | 99 (9.6) | 47 (4.6) |  |
| **JIA category,** no. (%)  RF-positive polyarthritis  RF-negative polyarthritis  Systemic JIA  Persistent oligoarthritis  Extended oligoarthritis  Psoriatic arthritis  Enthesitis-related arthritis | 22 (50.0)  139 (58.9)  18 (56.3)  186 (65.5)  100 (62.1)  48 (61.5)  89 (59.3) | 14 (31.8)  58 (24.6)  10 (31.3)  66 (23.2)  34 (21.1)  19 (24.4)  42 (28.0) | 4 (9.1)  22 (9.3)  4 (12.5)  20 (7.0)  21 (13.0)  9 (11.5)  17 (11.3) | 4 (9.1)  17 (7.2)  0 (0)  12 (4.2)  6 (3.7)  2 (2.6)  2 (1.3) |  |

**Table S3**. Symptom severity for depression and anxiety by JIA category (conventional cut-offs)

Legend: PHQ-9 (score 0-27), Patient Health Questionnaire-9; GAD-7 (score 0-21), Generalized Anxiety Disorder Scale-7.

**Table S4**. Further contributing centres

Josefinum Krankenhaus, Augsburg; Kinderrheumatologische Privatpraxis, Bensheim; Vivantes Klinikum Friedrichshain, Berlin; Landeskrankenhaus Bregenz; Vestische Kinder- und Jugendklinik Datteln; Städtisches Klinikum Dresden-Neustadt; Med. Einrichtungen der Heinrich-Heine-Universität Düsseldorf; Universitätsklinikum Erlangen; Kinderarztpraxis Haller, Gundelfingen; Universitätsklinikum Freiburg; Kinderarztpraxis Nimtz-Talaska, Frankfurt/Oder; Kinderarztpraxis Rühlmann, Göttingen; Martin-Luther-Universität Halle-Wittenberg; Universitätsklinikum Heidelberg; Kinderarztpraxis Lutz, Heidelberg; Kinder- und Jugendärzte Hürth-Park, Hürth; Universitätsklinikum Jena; Universitätsklinikum Schleswig-Holstein, Kiel; Kinderkrankenhaus der Stadt Köln; Helios Klinikum Krefeld; Kinder- und Jugendarztpraxis Maier, Leinfelden-Echterdingen; Städtisches Klinikum St. Georg, Leipzig; DRK Krankenhaus Lichtenstein, Lichtenstein; Johann-Gutenberg-Universität Mainz; Dr.-von-Haunersches Kinderspital der LMU, München; Universitätsklinik Münster; Kinder- und Jugendarztpraxis Gröbel, Paderborn; Helios Vogtland-Klinikum Plauen; Kinder- und Jugendarztpraxis Leipold, Regensburg; Elisabeth-Krankenhaus Rheydt, Mönchengladbach; Knappschaftsklinikum Saar, Püttlingen; Asklepios Kinderklinik Sankt Augustin; Diakonie-Klinikum Schwäbisch Hall; Olgahospital Stuttgart; Universitätsklinikum Ulm; Marien-Hospital Witten; Universitätsklinikum Würzburg.
